# Two new species of *Siphocampylus* (Campanulaceae, Lobelioideae) from the Central Andes

**DOI:** 10.3897/phytokeys.58.6973

**Published:** 2016-01-12

**Authors:** Laura P. Lagomarsino, Daniel Santamaría-Aguilar

**Affiliations:** 1 Department of Organismic and Evolutionary Biology, 22 Divinity Avenue, Harvard University Herbaria, Cambridge, Massachusetts, 02138, USA; 2 Current address: Missouri Botanical Garden, P. O. Box 299, St. Louis, Missouri 63166, USA

**Keywords:** Andean biodiversity hotspot, Asterales, Bolivia, centropogonid clade, Peru, South America, taxonomy

## Abstract

Two species of *Siphocampylus* (Campanulaceae: Lobelioideae) from the Central Andes of Peru and Bolivia are described, illustrated, and discussed with reference to related species. One species, *Siphocampylus antonellii*, is endemic to high elevation grasslands of Calca, Peru, while the second, *Siphocampylus siberiensis*, is endemic to cloud forests of Cochabamba, Bolivia. Both species are robust shrubs that produce tubular pink flowers that are likely pollinated by hummingbirds.

## Introduction

While the subfamily Lobelioideae Burnett of Campanulaceae Juss. is cosmopolitan in its distribution, more than half of its ~1200 species are restricted to the Neotropics (Lammers 2007). Most of these species (~550/680) belong to the Andean-centered centropogonid clade, which comprises *Burmeistera* Triana (~120 species), *Centropogon* C. Presl (~210 species), and the mainland species of *Siphocampylus* Pohl (~220 species). This clade is unique within Lobelioideae for its combination of Neotropical distribution, woody habit, entire corolla tube (i.e., neither fenestrate nor dorsally cleft), and floral adaptation to pollination by either hummingbirds or nectar bats (Lagomarsino et al. 2014; Lammers 2002; Muchhala and Lammers 2005). Within the centropogonid clade, *Siphocampylus* can further be distinguished by its capsular (vs. baccate) fruit. The genus, however, is not monophyletic (Antonelli 2008; Knox et al. 2008): in addition to the Caribbean species, which are distantly related to the centropogonid clade, its mainland species form at least 11 subclades that are polyphyletic with respect to the berry-producing *Centropogon* (Lagomarsino et al. 2014). This result is not surprising given the high degree of character overlap between *Centropogon* and *Siphocampylus*, which has frequently resulted in the incorrect filing of material in herbaria (Gleason 1921; Lammers 1998) and the occasional description of a new species to the incorrect genus or of uncertain placement (e.g., *Centropogon dubius* [Zahlbr.] E. Wimm. for both scenarios) (Gleason 1921). The non-monophly of these genera is largely due to the dynamic evolution of fruit-type within the clade: fleshy fruits have evolved approximately eight times from dry-fruited ancestors, likely as a result of migration to densely forested habitats (Givnish et al. 2005, 2009; Lagomarsino et al. 2014). While further phylogenetic information is necessary to begin to re-delimit generic boundaries to reflect evolutionary relationships within Neotropical Lobelioideae, *Siphocampylus* species are easily identified to genus within the confines of the current classification system, especially when fruiting material is available. Despite its non-monophyly, *Siphocampylus* remains a conspicuous, if poorly studied, component of the cloud forests and high elevation grasslands that comprise much of the tropical Andean global biodiversity hotspot (Myers et al. 2000). Future taxonomic and phylogenetic work focused on the centropogonid clade will undoubtedly result in the description of many new species and discovery of new clades defined by synapomorphies.

Here we describe two new species of hummingbird-pollinated *Siphocampylus*. Type specimens for both species were included in the most recent molecular phylogeny of the centropogonid clade, which is based on five plastid markers and includes relatively dense taxon sampling that spans all recognized taxonomic divisions, geographical occurrences, and morphological variation within Neotropical Lobelioideae (Lagomarsino et al. 2014). The phylogeny provides the information necessary to discuss these new species in relation to their closest evolutionary relatives. We additionally discuss similarities and differences of the species placed closely to their relatives in the most recent monograph of *Siphocampylus* (Wimmer 1943, 1953, 1968).

## Methods

Field collections focused on Campanulaceae were conducted in Bolivia in November–December, 2011 and in Peru in November–December, 2012. Species identification and description of new species resulting from this fieldwork utilized many taxonomic references (Gleason 1921; Lammers 1998; León and Lammers 2006; Wimmer 1937, 1943, 1953, 1968) and collections at the following herbaria: A, BOLV, GB, GH, LPB, MO, MOL, NY, USM, and USZ. Herbarium acronyms here and throughout follow the *Index Herbariorum* (Thiers 2013 [continuously updated]).

## Taxonomic treatment

### Siphocampylus antonellii

Taxon classificationPlantaeAsteralesCampanulaceae

Lagom. & D. Santam. sp. nov.

urn:lsid:ipni.org:names:77151883-1

Figs 1 , 2

#### Diagnosis.

*Siphocampylus antonellii* is similar to *Siphocampylus elfriedii*, but differs in its smaller, linear-oblanceolate leaves, ventral corolla lobe >1.3 cm long, and pleasant, lemon-like odor emitted from living plants.

#### Type.

Peru. Cusco: Calca, Lares, Calle entre Amaparaes y Suyo, Arriba de Amaparaes, 12°58'902"S, 077°50'W, 3799 m, 10 December 2012 (fl), *L. Lagomarsino*, *D. Santamaría, J. Wells, F. Farro 400* (holotype: A!; isotypes: GB!, MO!, NY!).

Shrub 1.5 m tall, branching 20 cm above the base, with soft wood; branches 0.2–0.6 cm in diameter, solid and fistulose, light brown to reddish purple in living material, glabrescent or white-tomentose; internodes 0.2–1.0 cm long; latex white. Leaves spirally arranged, distributed evenly along the branches, producing lemon-like odor; petiole 0.1–0.5 cm long, sometimes subsessile, villous, the trichomes whitish, adaxially canaliculate, abaxially rounded to triangular; lamina 3.8–5.2 × 0.3–0.55 cm, linear-oblanceolate, not rugose, appearing glabrous but densely pubescent with diminutive, whitish, stellate to echinoid trichomes; base attenuate to decurrent, sometimes with uneven sides; apex acuminate; margin sinuate, subentire, or diminutively dentate, 11–25 teeth per side, rounded to uncinate, sometimes appearing as a glandular callosity; venation reticulate, with 5–10 pairs of lateral nerves, ascending, impressed or indistinct adaxially, flat abaxially. Flowers solitary, axillary, generally towards the apex of branch; pedicel 3.0–5.0 cm long, straight for almost the entire length, but curved below the hypanthium, cylindrical or flattened, densely pubescent, bibracteolate; calyx lobes 5, 0.7–1.6 × 0.1–0.12 cm, linear-oblanceolate, margins diminutively dentate with 3–5 teeth per side, densely pubescent on both surfaces, straight, the apex acuminate; corolla (2.8–) 3.9–5.1 cm long, tube pink with yellow to light green lines parallel to the lobes, lobes light green-yellow externally, light yellow to cream colored inside, completely pubescent externally, pubescent internally with stellate to echinoid trichomes; tube 2.7–3.5 × 0.4–0.6 cm, constricted at the base and widening distally, straight at anthesis; corolla lobes 5, lanceolate to narrowly triangular, apex acute to acuminate, the two dorsal lobes 1.0–1.6 cm long, the two lateral lobes 1.3–1.5 cm long, the ventral lobe 1.4–1.9 cm long; staminal tube 3.5–4.5 × 0.1 cm, straight, glabrous, cream-colored to light green in living material, exserted between the two dorsal lobes; anther tube 0.5–0.7 × 0.2–0.21 cm, dark gray, glabrous, ventral anthers 0.4–0.6 cm long, penicillate at the apex, the trichomes white or yellowish gold, dorsal anther 0.45–0.6 cm long, glabrous. Fruits not seen.

#### Distribution and habitat.

*Siphocampylus antonellii* is endemic to Peru, where it grows on rocky slopes in puna habitat at ~3800 m in elevation. It is only known from the type collection.

#### Phenology.

Individuals were collected in flower in December; the rest of the phenology of this species remains unknown.

#### Etymology.

It is an honor to name this attractive species for Dr. Alexandre Antonelli (1978–), a biogeographer and phylogeneticist at the University of Gothenburg. Antonelli has made many important contributions to our understanding of Neotropical biodiversity through space and time, and to the evolution of various taxa, including Lobelioideae. His efforts in the latter brought the second author to the field in Costa Rica in 2005 in search of Campanulaceae, and helped to inspire the first author to study *Centropogon*, *Siphocampylus*, and *Burmeistera*.

#### Conservation status.

*Siphocampylus antonellii* is endemic to a narrow stretch of high-elevation grassland (puna) in Calca, Peru, where it is locally abundant. Only a single population of this species is known, from which the type collection was made. Due to its small area of occurrence and the threat of future deforestation in its habitat, we tentatively consider this species to be Vulnerable (IUCN 2014). Its vulnerable status is further justified by its roadside occurrence, near major construction efforts.

#### Discussion.

**Figure 1.**
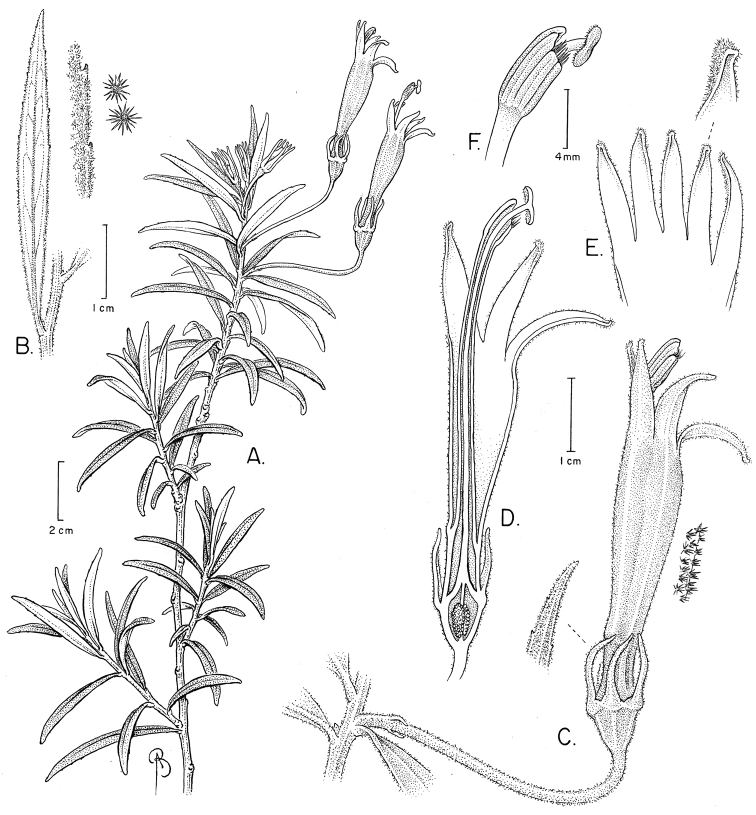
Siphocampylus antonellii. **A** Flowering branch **B** Leaf, abaxially, including detail of leaf margin and stellate hairs that cover surface **C** Staminate-phase flower, including bibracteolate pedicel, with detail of sepal and stellate hairs that cover the outer corolla surface **D** Longitudinal section of a pistillate-phase flower, showing the insertion of staminal tube to corolla, style and stigma as situated relative to the stamens, and bilocular ovary with axile placentation **E** Corolla lobe detail **F** Detail of anther tube, including apical hairs on ventral anthers, and stigma. Drawing by Bobbi Angel from the type.

**Figure 2.**
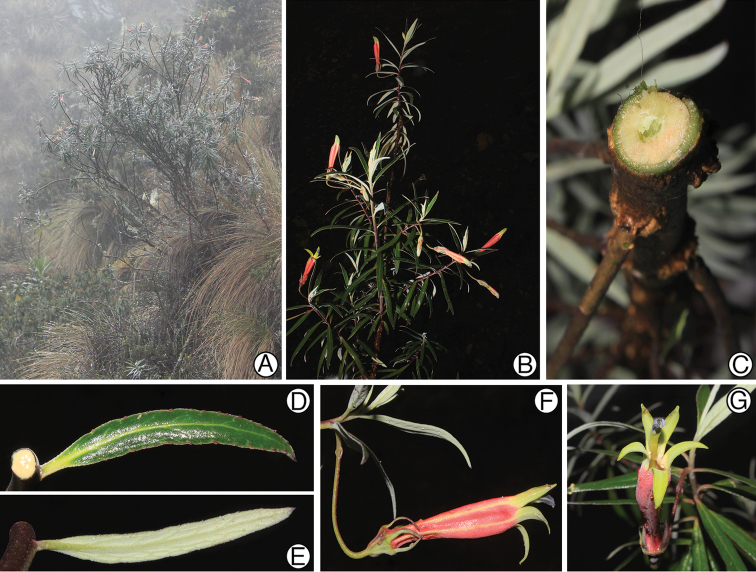
Siphocampylus antonellii. **A** Habit and high-elevation grassland (puna) habitat **B** Flowering branch **C** Cross-section of stem showing woody habit **D** Adaxial leaf surface **E** Abaxial leaf surface **F** Lateral view of flower in staminate phase **G** Anterior view of a flower, showing corolla aperture. All photos of the type collection, taken in the field by L. Lagomarsino.

**Figure 3.**
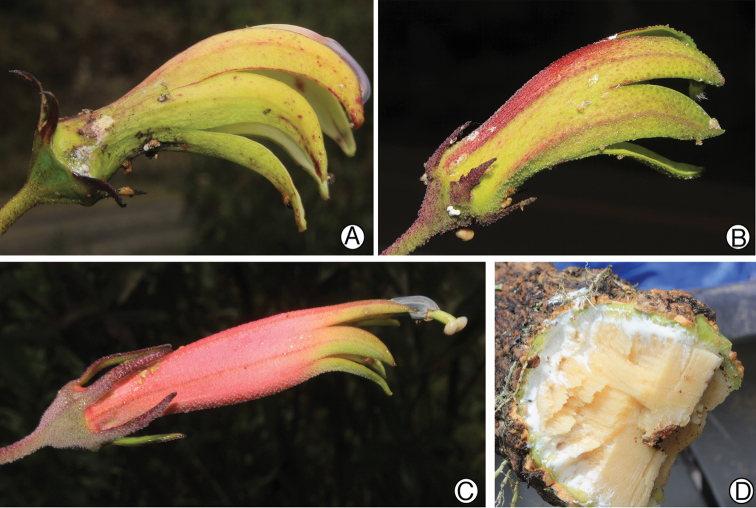
Closest relatives of *Siphocampylus antonellii*, based on molecular phylogeny of Lagomarsino et al. (2014). **A** Flower of *Siphocampylus veteranus* **B** Flower of *Siphocampylus actinothrix* **C** Flower of *Siphocampylus elfriedii* **D** Cross-section of main stem of *Siphocampylus veteranus*, showing a much more robust habit than *Siphocampylus antonellii*, but similar wood structure. All photos taken in the field by L. Lagomarsino. **A**, **D** *L. Lagomarsino et al. 388* **B** *L. Lagomarsino et al. 403* **C** *L. Lagomarsino et al. 387*.

*Siphocampylus antonellii* is most similar to *Siphocampylus elfriedii* E. Wimm. (Fig. 3C) and *Siphocampylus parvifolius* E. Wimm., which are both also endemic to Peru. These species share a shrubby habit (Fig. 2A), generally high elevation occurrence ([1050–] 2100–3800 m), bibracteolate pedicels (Figs 1C, 2F), pink or pinkish-purple corolla tubes with yellow-green lobes (Figs 2F–G, 3C), glabrous anthers (except at the apex of the ventral anthers) (Figs 1F, 3C), and turbinate ovaries (Figs 1C, 2F, 3C). Both *Siphocampylus antonellii* and *Siphocampylus elfriedii* produce echinoid trichomes, following Batterman and Lammers 2004. However, *Siphocampylus antonellii* can be easily distinguished by the differences enumerated in Table 1 and by the agreeable lemon-like smell that it emits.

**Table 1.** Differences between *Siphocampylus antonellii*, *Siphocampylus elfriedii*, and *Siphocampylus parvifolius*.

	*Siphocampylus antonellii*	*Siphocampylus elfriedii*	*Siphocampylus parvifolius*
Leaf shape	Linear-oblanceolate	Lanceolate	Lanceolate to oblanceolate
Leaf size	3.8–5.2 × 0.3–0.55 cm	5.0–7.4 × 1.1–1.9 cm	2.0 × 0.5 cm
Leaf margin	Margin sinuate, subentire, or diminutively dentate	Denticulate	Weakly crenulate
Sepal length	0.7–1.6 cm	0.7–1.4 cm	0.3 cm long
Sepal margin	Diminutively dentate	Denticulate	Entire or diminutively denticulate
Corolla length	(2.8–) 3.9–5.1 cm	3.9–4.6 cm	3.4 cm
Corolla indument	Pubescent	Pubescent	Glabrous
Dorsal corolla lobe length	1.0–1.6 cm	0.9–1.2 cm	1.1–1.4 cm
Ventral corolla lobe length	1.4–1.9 cm	0.5–1 cm	0.9–1.2 cm
Reference sheets	*L. Lagomarsino 400* (A)	*L. Lagomarsino* 387 (GH); *J. L. Luteyn & M. L. Lebrón-Luteyn 6395* (MO); *F. W. Pennell 13841* (GH); *T. Plowman & E. W. Davis 5126* (GH); *J. West 7045* (GH)	*R. D. Metcalf 30469* (A)

This new species would be placed near *Siphocampylus nobilis* E. Wimm. and *Siphocampylus rosmarinifolius* G. Don in the dichotomous key of genus in Wimmer (1953). In addition to being restricted to Peru, these species share linear leaves, turbinate ovaries, and narrow corolla lobes. However, these two species have glabrous sepals and corolla (vs. pubescent in *Siphocampylus antonellii*), ebracteolate, glabrous pedicels (vs. bibracteolate and pubescent), and different corolla colors: reddish orange with yellow lobes in *Siphocampylus rosmarinifolius* and purple in *Siphocampylus nobilis* (vs. pink with yellow-green lobes [Fig. 2F]). *Siphocampylus nobilis* can be further distinguished from *Siphocampylus antonellii* by its vining habit (vs. shrubby), long, narrow leaves (6.0–8.0 × 0.2–0.3 vs. 3.8–5.2 × 0.3–0.55 cm), and glabrous branches, leaves, pedicels, and hypanthium (vs. pubescent [Fig. 1]). *Siphocampylus rosmarinfolius* can be further distinguished by its longer leaves (8.0–11.5 cm vs. 3.8–5.2 cm), the rugose, glabrous adaxial leaf surface (vs. not rugose and pubescent), and revolute leaf margins (vs. not revolute).

Molecular phylogenetic analysis places *Siphocampylus antonellii* in a well-supported clade that includes *Siphocampylus actinotrix* E. Wimm. (Fig. 3B), *Siphocampylus elfriedii* (Fig. 3C), *Siphocampylus vatkeanus* Zahlbr., *Siphocampylus veteranus* E. Wimm. (Fig. 3A, D), and *Siphocampylus rictus* Lammers; within this clade, *Siphocampylus antonellii* is sister to *Siphocampylus veteranus* (Lagomarsino et al. 2014). This clade as a whole is composed of generally tall, woody shrubs. The placement of *Siphocampylus elfriedii* in this clade is not surprising, as it is quite similar to *Siphocampylus antonellii* (see above). However, it is more difficult to find similarities with the remaining species, which can be easily distinguished from *Siphocampylus antonellii* by a series of putative adaptations to pollination by bats: long pedicels (7.0–15 cm), wide corolla apertures, and longer corollas (3.2–5.0 cm) that are generally dull in color (either green, cream-colored, white or yellowish, sometimes mottled with reddish pigmentation) (Fig. 3A–B). Additionally, these species have longer leaves (6.0–13.5 cm vs. 3.8–5.2 cm) and a glabrous or only sparsely pubescent corolla (but with echinoid-stellate pubescence in *Siphocampylus actinothrix* [Fig. 3B]). Despite the similarities discussed above, *Siphocampylus rosmarinifolius* falls outside of the clade that includes *Siphocampylus antonellii* (Lagomarsino et al. 2014); *Siphocampylus nobilis* and *Siphocampylus parvifolius* have not yet been sampled in a molecular phylogenetic analysis.

### Siphocampylus siberiensis

Taxon classificationPlantaeAsteralesCampanulaceae

Lagom. & D. Santam. sp. nov.

urn:lsid:ipni.org:names:77151884-1

Figs 4 , 5

#### Diagnosis.

*Siphocampylus siberiensis* is similar to *Siphocampylus boliviensis*, but with a corolla tube lacking a constriction at its base and a hemispherical hypanthium.

#### Type.

Bolivia. Cochabamba: Carrasco, en la entrada para Sunchal, cerca al rótulo “Unidad Educativa Manuela Gandarillas”, 17°47'267"S, 064°47'669"W, 2668 m, 18 December 2011 (fl & fr), *L. Lagomarsino, D. Santamaría & J. M. Mendoza 241* (holotype: A!; isotype: LPB, USZ).

Multi-stemmed shrub 3–4 m tall, branched, all branches arising from a single point at ground level, with soft wood, the bark suberose; branches 0.4–0.6 cm in diameter, fistulose, the youngest parts purple, brown when mature (greyish to whitish in dry material), glabrescent to tomentose; internodes 0.9–1.7 cm long; latex white. Leaves spirally arranged and generally clustered at the apex of branches, leaving prominent leaf scar after falling; petiole 0.3–0.7 cm long, glabrescent to tomentose with whitish trichomes, winged, adaxially canaliculate, abaxially more or less triangular with two ribs; leaf blade 10.5–19.5 × 2.9–4.6 cm, oblanceolate, adaxially tomentose and abaxially densely pubescent, the pubescence principally on the veins, trichomes simple, the base decurrent; apex acuminate; margin doubly dentate and ciliate, 75–95+ teeth per side, the teeth triangular; venation reticulate with 16–21 pairs of lateral nerves, lightly ascendant, impressed adaxially and elevated abaxially. Flowers solitary, axillary, generally towards branch apex; pedicel 4.5–9.4 cm long, straight, cylindrical, densely pubescent; bracteoles absent; hypanthium 0.5–0.8 × 0.3–0.4 cm, hemispherical, tomentose; calyx lobes 5, (0.8–) 1.0–1.4 × (0.2–) 0.3 cm, narrowly triangular, the margins ciliate, entire, pubescent on both surfaces, erect or recurved, the apex acuminate; corolla 3.5–3.8 cm long, completely pink, diminutively pubescent on both surfaces; corolla tube 0.9–2.2 × 0.7–0.8 cm, cylindrical for its entire length, a little wider apically than basally, straight at anthesis; corolla lobes 5, narrowly triangular, slightly falcate, the margins ciliate, the apex acute to acuminate, the two dorsal lobes 1.5 cm long, the two lateral lobes 1.4 cm long, the ventral lobe 1.4 cm long, staminal tube 3.0–3.8 × 0.1–0.2 cm, straight, sparsely pubescent, pink, exserted between the two dorsal lobes; anther tube 0.8–0.9 × 0.2–0.3 cm, gray in living material, glabrous except in the sutures between anthers, which are densely pubescent, the trichomes white, ventral anthers 0.6–0.8 cm long, penicillate at the apex, the trichomes white, the dorsal anthers 0.7–1.0 cm long, penicillate at the apex, the trichomes white. Fruit capsule, 5.0 × 1.0–1.2 cm, ca. 15-lobed, with external ridges, the calyx persistent; seeds not seen.

#### Distribution and habitat.

*Siphocampylus siberiensis* is endemic to Bolivia, where it has been collected at the edge of the road at ca. 2700–2900 m in elevation in cloud forest.

#### Phenology.

Individuals were collected in flower and fruit in December and in flower only in April; the rest of the phenology of this species remains unknown.

#### Etymology.

The specific epithet of this species refers to the type locality, the Serranía de Siberia, a mountain range at the limit between the Cochabamba and Santa Cruz departments in Bolivia.

#### Conservation status.

*Siphocampylus siberiensis* is known only from a single population in Serranía de Siberia in central Bolivia; this population is represented by the two cited collections. This species appears to be locally rare, and only one individual was encountered during our fieldwork. Due to its small area of occurrence and the threat of future deforestation in its habitat, we tentatively consider this species to be Vulnerable (IUCN 2014). Its vulnerable status is further justified by its roadside occurrence in montane cloud forest, a habitat type known to be particularly sensitive to human encroachment.

#### Discussion.

**Figure 4.**
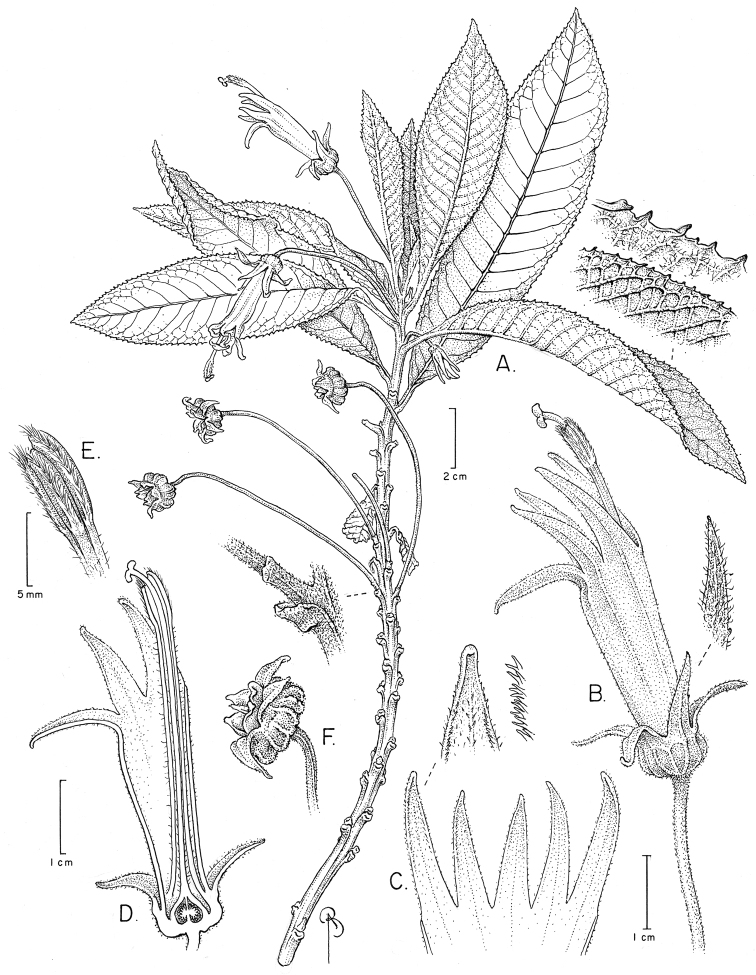
Siphocampylus siberiensis. **A** Flowering branch, showing the persistent leaf scars and developmental procession of distal flower at anthesis to basal capsular fruit **B** Flower in pistillate phase, including detail of sepal with pubescence **C** Corolla lobe detail, including marginal pubescence **D** Longitudinal section of a pistillate phase flower, showing the insertion of staminal tube to corolla, style and stigma as situated relative to the stamens, and bilocular ovary with axile placentation **E** Anther tube in staminate-phase flower **F** Capsule with lobes and ridges. Drawing by Bobbi Angel from the type.

**Figure 5.**
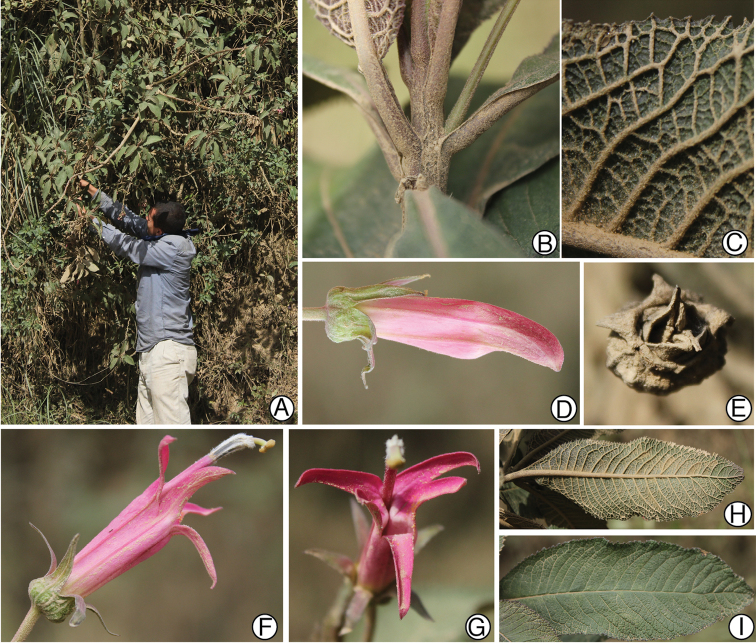
Siphocampylus siberiensis. **A** Habit **B** Detail of young stem **C** Detail of leaf margin and venation on abaxial leaf surface **D** Flower bud **E** Capsule **F** Lateral view of flower in pistillate phase **G** Anterior view of corolla, showing corolla aperture **H** Abaxial leaf surface **I** Adaxial leaf surface. All photos of the type collection, taken in the field by L. Lagomarsino; D. Santamaría-Aguilar is shown collecting the type in **A**.

*Siphocampylus siberiensis* can be recognized by its shrubby habit with multiple stems arising from a single point; leaves aggregated at the apex of branches (Fig. 4A); conspicuous venation (especially on the abaxial leaf surface) (Figs 4A, 5C); solitary flowers borne in the axil of leaves (Fig. 4A); light pink corolla with a tube that is cylindrical for its entire length (i.e., not basally constricted) (Figs 4B, D, 5F); anther tube that is densely pubescent in the sutures between individual anthers (Figs 4E, 5F); and fruits that are both ribbed and lobed (Figs 4F, 5E).

**Figure 6.**
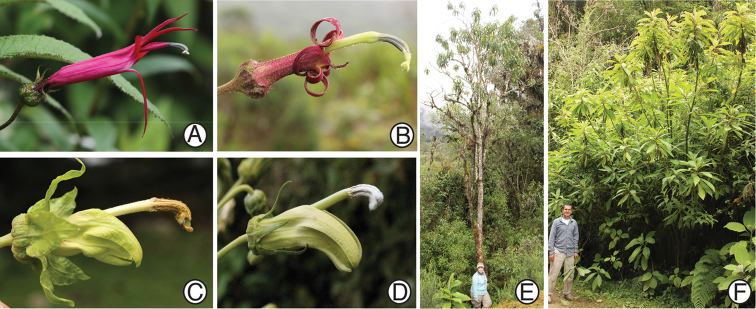
Closest relatives of *Siphocampylus siberiensis*, based on molecular phylogeny of Lagomarsino et al. (2014). **A** Flower of *Siphocampylus boliviensis* **B** Flower of *Siphocampylus tunarensis* **C** Flower of *Siphocampylus tunicatus* **D** Flower of *Siphocampylus umbellatus* **E** Habit of *Siphocampylus tunarensis*, shown with L. Lagomarsino **F** Habit of *Siphocampylus tunicatus*, shown with D. Santamaría-Aguilar. All photos taken in the field by L. Lagomarsino (**A–D, F**) and D. Santamaría-Aguilar (**E**). **A** *L. Lagomarsino et al. 239* **B, E** *L. Lagomarsino et al. 232* **C, F** *L. Lagomarsino et al. 235* **D** *L. Lagomarsino et al. 193*.

Molecular phylogenetic analysis places *Siphocampylus siberiensis* in a clade that includes *Siphocampylus tunarensis* Zahlbr., *Siphocampylus tunicatus* Zahlbr., and *Siphocampylus umbellatus* (Kunth) G. Don; this clade is closely related to *Siphocampylus boliviensis* Zahlbr. and *Siphocampylus sparsipilus* E. Wimm. (Lagomarsino et al. 2014) (Figs 5, 6). These species are all restricted to the Central Andes of Peru and Bolivia, with the exception of *Siphocampylus umbellatus*, whose range also extends to Brazil. This clade is composed of robust shrubs or trees that are exceptionally tall for the centropogonid clade (Figs 5A, 6E–F), or rarely scandent subshrubs (*Siphocampylus sparsipilus* and some collections of *Siphocampylus boliviensis*), with ebracteolate pedicels, a shallow, hemispherical hypanthium (turbinate in *Siphocampylus tunarensis*), and leaves that leave prominent scars after abscission (Fig. 4A) and have dentate margins and reticulate venation that is conspicuous on both surfaces, but especially the abaxial surface (Figs 4A, 5C). Both bright pink (*Siphocampylus siberiensis*, *Siphocampylus boliviensis*, *Siphocampylus sparsipilus*) and dull colored (*Siphocampylus tunarensis*, *Siphocampylus tunicatus*, *Siphocampylus umbellatus*) corollas are represented in this clade. This color variation, which is associated with different gross corolla morphologies, likely reflects adaptation to different pollinators (hummingbirds and bats, respectively) (Figs 5F–G, 6A–D).

Even though they are not the most closely related species, the pink, narrow flowers of *Siphocampylus siberiensis* most closely resemble those of *Siphocampylus boliviensis* and *Siphocampylus sparsipilus*. However, the latter two species can be easily distinguished by their corollas that are constricted at the base (vs. not constricted) and much rounder hypanthium (vs. flattened at top). The other species in the immediate clade that includes *Siphocampylus siberiensis* differ in their dull reddish (*Siphocampylus tunarensis* [Fig. 6B]) or whitish-green (*Siphocampylus tunicatus* [Fig. 6C], *Siphocampylus umbellatus* [Fig. 6D]) corollas (vs. bright pink in *Siphocampylus siberiensis*). *Siphocampylus tunicatus* and *Siphocampylus siberiensis* are sister species that are vegetatively very similar, though their flowers are markedly different (Figs 4, 5, 6C, F). In addition to its green corolla, the former can be distinguished by its longer sepals (2.0–2.8 cm [Fig. 6C] vs. [0.8–] 1.0–1.4 cm [Figs 4B, D, 5F]) that are leaf-like (vs. not leaf-like) and its wider hypanthium (1.5–2.0 vs. 0.3–0.4 cm) (Fig. 6C). *Siphocampylus tunarensis* can be separated by its linear, revolute corolla lobes (Fig. 6B) (vs. narrowly triangular and not revolute [Figs 4B, 5F–G]) and short sepals (0.2–0.4 cm [Fig. 6B] vs. 1.0–1.4 cm [Fig. 5F]). Furthermore, while *Siphocampylus siberiensis* is a robust shrub 3–4 m tall, *Siphocampylus tunarensis* can grow to be a very tall tree (>10 m) with a diameter of more than 30 cm and is possibly one of the largest species of Campanulaceae in the Americas (Fig. 6E).

The species that is most superficially similar to *Siphocampylus siberiensis*, *Siphocampylus boliviensis*, is placed in the same couplet as *Siphocampylus macrostemon* A. DC. in the dichotomous key to the members of the genus in Wimmer (1953). This markedly different species, which has not yet been sampled in molecular phylogenetic analysis, can be distinguished by its subsessile leaves (vs. pedicels 0.3–0.7 cm long) that are smaller (5–8 × 1.31.5 cm vs. 10.5–19.5 × 2.9–4.6 cm) and sparsely pubescent on the adaxial surface (vs. tomentose), minutely dentate leaf margins (vs. doubly dentate and ciliate), shorter pedicels (2.6–4.2 cm vs. 4.5–9.4 cm) that are bracteolate (vs. ebracteolate), and glabrous corolla (vs. pubescent). The other species most closely related to *Siphocampylus siberiensis* fall into many disparate taxonomic units within the current classification scheme of the genus. This makes it difficult to place this new species in the context of Wimmer’s taxonomy; this is likely due to this treatment’s reliance on single, often arbitrary characters to designate groups.

The measurements of the calyx and corolla in parentheses correspond to *E. Fernández et al. 3583* (MO). This specimen apparently has a white corolla, but otherwise corresponds to the species concept for *Siphocampylus sibieriensis* presented here.

#### Additional specimens examined.

Bolivia: Cochabamba, Carrasco, Siberia, 17°48'11"S, 064°46'12"W, 2900 m, 16 April 2005 (fl), *E. Fernández et al. 3583* (MO).

## Supplementary Material

XML Treatment for Siphocampylus antonellii

XML Treatment for Siphocampylus siberiensis
